# Ontology-based, Tissue MicroArray oriented, image centered tissue bank

**DOI:** 10.1186/1471-2105-9-S4-S4

**Published:** 2008-04-25

**Authors:** Federica Viti, Ivan Merelli, Andrea Caprera, Barbara Lazzari, Alessandra Stella, Luciano Milanesi

**Affiliations:** 1 Istituto di Tecnologie Biomediche – Consiglio Nazionale delle Ricerche, Segrate (Milan) 20090, Italy; 2 Parco Tecnologico Padano, Cascina Codazza (Lodi) 26900, Italy; 3 Biolab - Department of Computer Science, Control Systems and Telecommunications (DIST) - University of Genoa, Genoa 16100, Italy

## Abstract

**Background:**

Tissue MicroArray technique is becoming increasingly important in pathology for the validation of experimental data from transcriptomic analysis. This approach produces many images which need to be properly managed, if possible with an infrastructure able to support tissue sharing between institutes. Moreover, the available frameworks oriented to Tissue MicroArray provide good storage for clinical patient, sample treatment and block construction information, but their utility is limited by the lack of data integration with biomolecular information.

**Results:**

In this work we propose a Tissue MicroArray web oriented system to support researchers in managing bio-samples and, through the use of ontologies, enables tissue sharing aimed at the design of Tissue MicroArray experiments and results evaluation. Indeed, our system provides ontological description both for pre-analysis tissue images and for post-process analysis image results, which is crucial for information exchange. Moreover, working on well-defined terms it is then possible to query web resources for literature articles to integrate both pathology and bioinformatics data.

**Conclusions:**

Using this system, users associate an ontology-based description to each image uploaded into the database and also integrate results with the ontological description of biosequences identified in every tissue. Moreover, it is possible to integrate the ontological description provided by the user with a full compliant gene ontology definition, enabling statistical studies about correlation between the analyzed pathology and the most commonly related biological processes.

## Background

One of the most promising biomolecular approaches of recent years in the cancer research field is the Tissue MicroArray (TMA) technique. A paraffined embedded TMA is a matrix of cores of fixed tissue samples: tissues come from *donor blocks* and are organized in a new paraffined *recipient block*. In this way pathologists or researchers may perform reactions in a parallel way on tens or hundreds of tissues at a time, with a decrease of experiment time and reagents costs, an improvement in normalization between samples, which are treated in a single reaction, and enabling the reuse of scarce resources such as tissue blocks [1]. The TMA technique deals with a huge number of images: first of all hematoxylin-eosin (HE) slides of donor block tissues, which represent *pre-array images*. Then, if we consider that from the elaboration of a single TMA block hundreds of slices are obtained, that these can be processed with different reagents, and that each slice of the block can contain hundreds of samples, we can prefigure the amount of single spot images, or *post-array images*, that an institute of pathology must handle.

Handling and mining all TMA data is extremely difficult without a suitable management system to deal with storage and data integration. To accomplish this task, a number of applications can be used. Some software tools are commercially available, like tools developed by SlidePath [2], for information and image analysis, and by Aperio [3], for digital pathology. In addition to not being freely downloadable, these solutions are less customizable and not very much flexible for ongoing experimental research. Therefore we examined a range of highly customizable open source tools for TMA management. Most of them rely on web interfaces on the top of local databases to store TMA related information, such as patients' clinical data, specimens, donor blocks, cores, and recipient blocks and in some cases also provide the possibility to elaborate microscope acquired images. An essential approach to TMA data management is provided by TAD [4], TmaDB [5] and TIMAN [6], which are well organized tools limited to TMA preparation, output submission and elaboration, and data queries.

Long Liu C. *et al*. propose an interesting application developed at Stanford University [7], which includes various possibilities to elaborate and analyze TMA outputs. This is a rich and useful platform focused on the post-array phase, but it lacks support for the experiment designing phase. More complete management tools come from Morgan JD *et al*., who propose the TMAJ [8] system, and Demichelis F *et al*., who developed TMABoost [9]. The former is a Java based software which allows the porting of interesting applications, including efficient image analysis algorithms and the importing of data tools. This platform is oriented not only to results sharing but also to automatic data submission: the user has the possibility of automatically importing formatted data, thus avoiding time-consuming and error-generating manual entering. On the other hand, TMABoost is probably the most advanced tool for supporting TMA experiments. Its peculiarity is the interdisciplinary approach, supported by the creation of different roles for users, associated to different permissions and competences. The system is deeply oriented to both the samples data submission and the output data elaboration and sharing.

Nevertheless, neither of these two systems allows deep interactions among pathologists and researchers either limiting intense tissue sharing and neglecting the idea to increase possibilities of improved TMAs creation by making thousands of samples stored in the anatomo-pathology institutes available to researchers. Moreover, there is a lack of data integration of clinical and biomedical information with bioinformatics knowledge, an approach that enriches the traditional histo-pathology vision, adding the molecular biology information to the histological and clinical data, and providing analysis methods based on statistics and data mining.

The aim of the work is to provide pathologist community a simplified tool to manage their experiments in order to facilitate data exchange and integration with other information available on line. The platform arises from the necessity, not solved by other existing platforms, to enrich the support given to TMA experiments, because in our opinion the communication between pathologists, researchers and bioinformaticians of different institutes is important for the spreading of this technique. Indeed, a peculiarity of the proposed system is the integration in the web application of a strong ontology mechanism to guarantee standardized descriptions for biological materials and experiment outputs. Moreover, the system provides high integration to molecular biology knowledge: although the technique is pretty close to the bioinformatics field, there are no references in other platforms about managing genomic, transcriptomic and proteomic data coming from TMA experiments. The described work contains a first attempt to provide statistics on the bioinformatics data, such as genes and proteins related to metabolic pathways.

## Methods

Our system relies on different components. Storage and data integration are provided by a MySQL RDBMS, which is useful for creating links between data associated with tissues and TMA experiments. Two ontology terms services were developed to integrate and improve information about cancer topic, one applied to pre-array HE treated tissue images and the other associated to post-array immunohistochemical and biomolecular reactions images. In both cases we used the ontology to provide semantic web integration with on line papers, in order to provide literature data related to the experiment.

In order to share data among the research community, a number of information must be associated to samples. Working on literature information [10] and developing the system in close collaboration with pathologists, we defined four groups of important data to support biological samples:

○ patients' clinical data;

○ sample origin, extraction and preservation;

○ TMA block aim and structure;

○ post-analysis image data.

**Figure 1:**
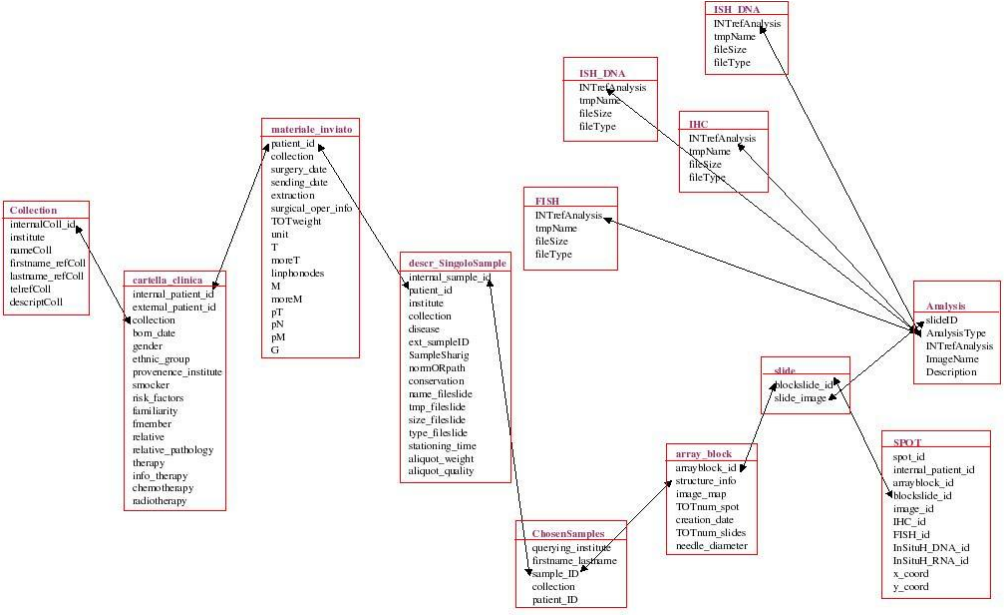
**Simplified version of the database schema.** The figure presents a simplified view of the tables, included in the MySQL TMA database, which store all interesting data of a TMA experiment.

The TMA database structure is presented in Figure 1. The schema includes tables concerning the case history of the patient, an accurate description of samples extracted from that patient, the TMA information and data related to the analysis made on tissue slices. The schema and the data access are completely transparent to the user through the web interface.

### Open Biomedical Ontology (OBO) and Lookup Service (OLS) Integration

In order to standardize descriptions we chose to use medical ontology terms. Ontology is a collection of terms naming descriptors in a hierarchical structure that allows searching at various levels of specificity in a particular domain. Ontologies generally describe *individuals*, which are the basic objects, *classes*, that are the categories which objects belong to, *attributes*, which are the features the objects can have, and *relations*, that are the ways objects can be related one another. Open Biomedical Ontology (OBO) [11] is a collection of well-structured controlled vocabularies for sharing information across different biological and medical domains. The site contains ontologies designed for different biomedical aspects: some of them are generic and can be applied across all organisms, while others are more restricted in scope, for example to specific taxonomic groups.

To easily handle the *obo* ontology format we integrated in our web site the open source Ontology Lookup Service (OLS) [12]. It provides a user-friendly single entry point for publicly available *obo* ontologies in a single database. It can be accessed interactively by users or through a specific client. Using the web interface, the database can be queried using a user-friendly auto-completion search mechanism, to look in the ontology for a single specific term. Otherwise it is possible to browse the complete ontology tree using AJAX library and the system can be queried using a standard SOAP web service described by a WSDL descriptor.

### Ontology for tissues and for genes

Following the traditional pathologists specification, the site provides the SNOMED classification [13], for terms related to disease and organism site, while image description relies on ontology terms. In the *pre-analysis* phase each image representing a slice of HE treated tissue is followed by a text description, to add precious information to the samples. This is important to better understand which kind of tissue is stored and if it can be useful for some researchers in designing their TMA experiments. To provide a complete overview of ontology terms correlated to TMA we also developed a *post-analysis* phase, concerning ontological classification of genes and proteins detected in the experiments.

We used the Medical Subject Headings (MeSH) ontology [14] to provide users with a vocabulary of scientific community-recognised terms. It works on the top of the National Library of Medicine's, developed by the National Institutes of Health (NIH) and it has been developed directly in the framework of PubMed. These features make MeSH one of the most used tools for semantic web in pathology: we considered it as the best reference for an ontology based vocabulary of medicine and we chose it over other existing ontology based semantic web tools like, for example, Textpresso or E-Bioscience. Moreover, it has an on-line web browsing service and it works with the *obo* format, which allows simple handling of ontology terms, independently from the MeSH web site.

**Figure 2:**
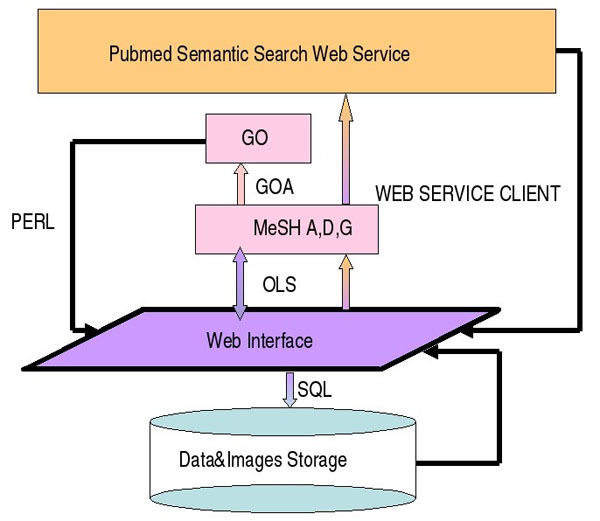
**System architecture**. From the web interface developed in html, php and javascript users can access the ontology term handler (based on MESH and GO ontologies) and the underlying RDBMS which contains sample and array data. The ontology terms allow users to perform a semantic web search to link litarature to the described concepts.

To provide standard descriptions in both pre and post analysis contexts, we installed the whole OLS platform uploading the MeSH ontology. Users can browse the subset A (Anatomy) for the tissue-related terms, and the subsets C - D12 - D06 - D08 - D13 - D23 (Diseases - Amino Acids, Peptides, and Proteins - Hormones - Enzymes - Nucleic Acids, Nucleotides, and Nucleosides - Biological Factors), for gene and protein representation. All these services are accessible from a web user interface as shown in Figure 2 that describes the organization of our system.

### GO statistical analysis

Considering that TMA experiments always aim to find the presence of proteins or genes in selected tissues, the possibility to integrate information about Gene Ontology (GO) [15] becomes very important. The extraction of statistical information about genes and proteins probed on TMA could also be very interesting when performing statistical analysis and providing information about metabolic pathways or processes involved in specific cancer diseases.

The user is asked to insert the gene or the protein probed on the TMA in a form. The value is sent to Entrez [16], the National Centre for Biotechnology Information (NCBI) tool for database interrogation, in the Gene and Protein sections, and the page is parsed to offer the user more detailed options to identify the used probe. We chose to query the Entrez search engine to guarantee an up-to-date database of genes and proteins without the need to create a mirror for local data. The Entrez Gene IDs or the UniProt IDs are extracted and related by the developed system to tables obtained from the GOA (Gene Ontology Association) file. In this way for each gene or protein searched for in a sample recorded in the database the system traces to what disease it is associated performing a statistical study of the most analyzed pathways in every pathology.

### Semantically linked articles

Another important issue of our ontology compliance TMA-oriented tissue bank is that the use of standardized descriptions provides an easy access to articles related to specific topics. In this way we can support literature mining, specific for each sample in the database, thus promoting data integration. In order to handle a semantic approach for finding related articles, we integrated a connection to MeSH database through NCBI Entrez in our system. Using this tool, the implemented system browses the MeSH ontology based classification for a semantic web search in both tissue-oriented pre-analysis and gene/protein post-analysis terms, to give users supplementary information about tissue histology and correlation between gene and pathology. Using the NCBI web service a properly formatted ontological query is sent to PubMed [17] in order to retrieve results, which are published papers concerning the topic related to the specific ontological term. The client has been implemented using a PHP Extension and Application Repository (PEAR) [18] module for SOAP communication.

## Results

A web interface has been developed to consistently access data stored in the database. The web site is implemented in PHP language, with the integration of PEAR library to improve functionality, while all the ontological aspects are managed by OLS. In order to provide a user-friendly system AJAX functions have been used for searching ontology terms even in the auto-completion terms mode. A crucial aspect the system takes care of is the security problem, considerably important in all biomedical applications dealing with sensitive patient information. To protect data in transit between the user's computer and the web server and vice-versa, we chose the HTTPS protocol, the secure HTTP connection for data transfer, which works just alike to HTTP but with a different default TCP port (443), and an additional encryption/authentication layer between the HTTP and TCP.

A very important aspect for security relies on the policy used to grant access credentials. Users must register previously to use our on-line support to the TMA oriented tissue bank in order to guarantee data security. Then, a specific role is assigned to each user according to his necessity:

○ *researcher*: has the possibility to see every sample inserted in the tissue bank and to browse all related information but can not insert any new sample;

○ *technician* or *pathologist*: is able to insert new samples or modify existing ones and can browse every sample belonging to the institute he works for;

○ *supervisor*: can insert or modify all samples belonging to his institute and can browse all the samples contained in the database.

**Figure 3:**
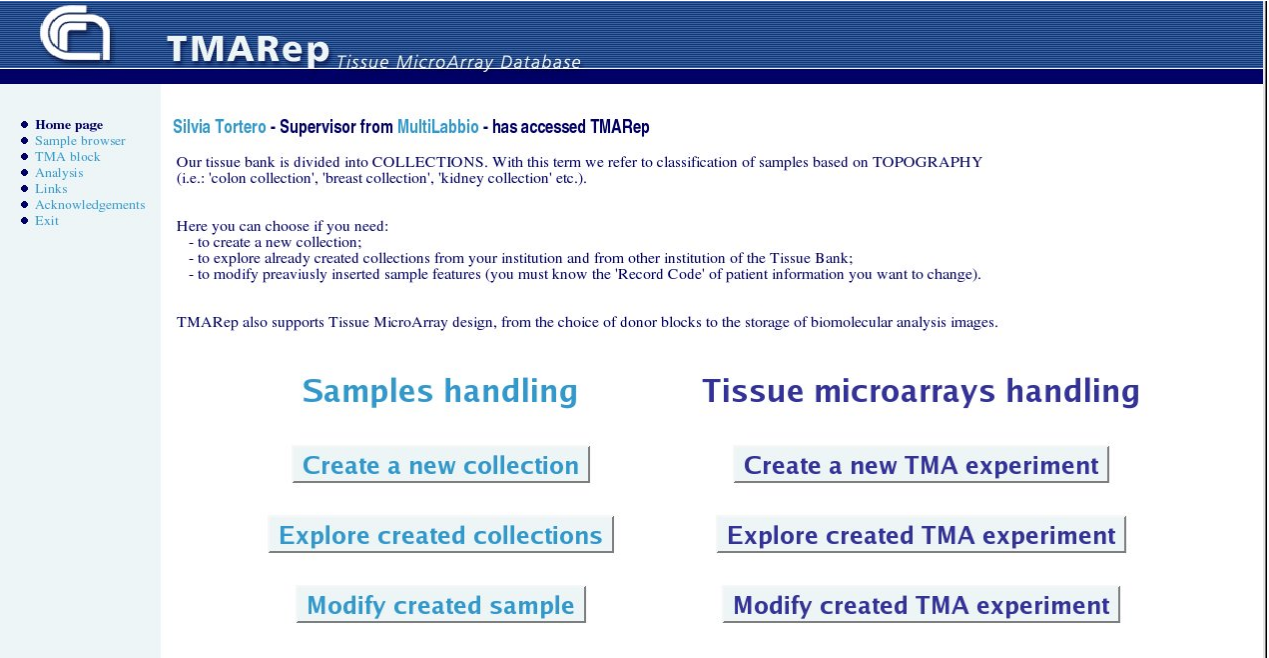
**Web site home page**. This is the home page of the TMARep site. Logged people are recognized and, depending on their role, can perform different actions relate to sample data uploading and visualizing or TMA building and analyzing.

According to the double aim of the site, both to provide a tissue data bank and to facilitate the creation of TMA blocks, users logged into the TMARep have access to samples and TMA experiment descriptions management according to their assigned role, as shown in Figure 3.

Samples belonging to each institute must be assigned to *collections*, which are groups of tissues referring to the same disease. This work is specifically oriented to cancer affected tissues, so every collection should be defined as a type of cancer. Users who want to upload a new sample firstly have to insert the patients' clinical data, then to complete the form about tissue extraction and localization information, specifying preservation data for every sample obtained from the patient. In this form users must also upload an image of the hematoxylin-eosin reaction made on the first slice of the paraffin embedded tissue.

A short description must be associated to every image, using suggested ontologically-compliant terms. The ontology based system is crucial to support tissue sharing and information integration. For example terms like “colorectal cancer” and “colon cancer” describe the same phenomena. If a pathologist uses the first expression while another prefers the second one, the associations of the two terms in a free-text based description system can be difficult. The problem has been solved thanks to the ontology approach, which suggests unique terms among which pathologists must chose. Moreover, pathologists can describe samples at different levels of details: a biopsy can be annotated as belonging to the “colon ascending” tract, while a shorter description could involve only the term “colon”. In this situation the ontology tree plays a crucial role in correlating these two terms, because the second is parent of the first one. Moreover, ontologies are useful for data integration from external resources: terms can be used to interact with the MeSH semantic web system to link the sample description with related articles reported in PubMed.

When a researcher browses the tissue bank and finds some interesting samples, he can tick them off in order to include them in his TMA experiments, according to their effective availability. To facilitate TMA experiment design, the web system provides a complete description of the selected samples which can be downloaded in a standard xls spreadsheet thanks to the integration in the web site of specific PEAR functions. This is very useful both for supporting the creation of the TMA, because it represents a summary of the characteristics of the samples, and for uploading results in the web site, in the post analysis phase.

**Figure 4:**
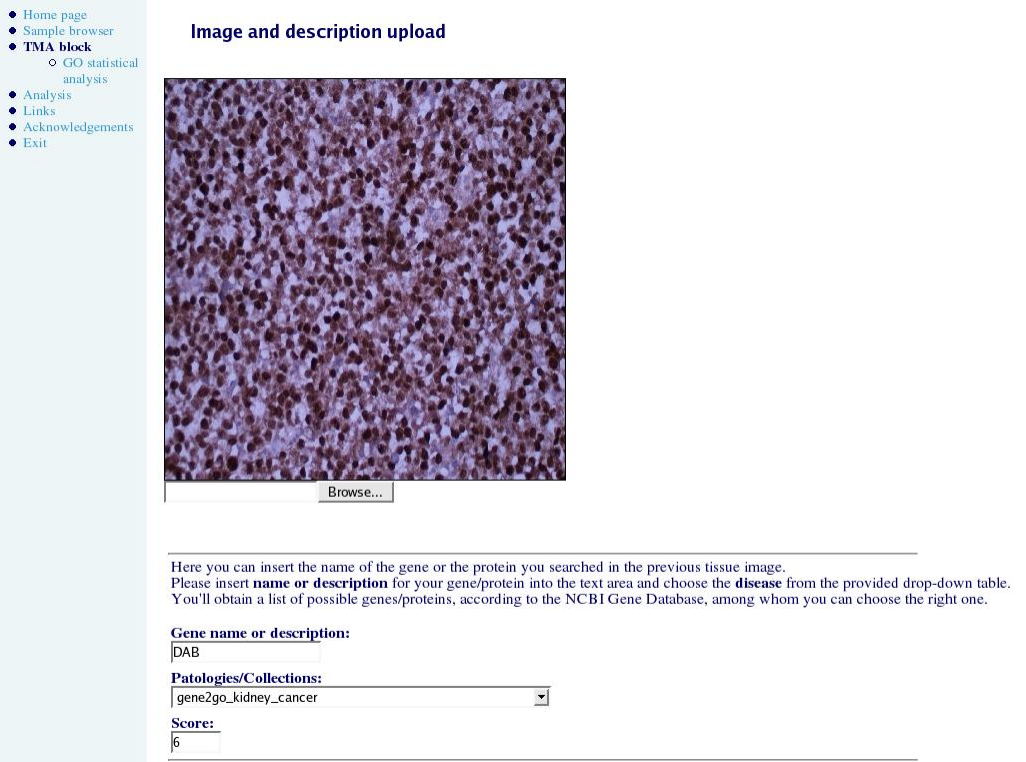
**Spot uploading web page**. This is an example of the TMARep site web page where the user is asked to upload spot image, gene target and reaction score.

After producing and processing TMA blocks, users can complete the dedicated form, explaining experiment aim and design, and uploading images of single spot reactions. This is an important step because the researcher is asked to report a quantification of the results obtained in his experiments: he is asked to associate to every processed spot the name of the gene or the protein that has been hybridised *in situ* and a score to quantify its presence in the spot, as shown in Figure 4. The grading methodology is based on the immunoreactive score (IRS) [19] definition: the total IRS range is 0-9 and has to be calculated multiplying the *staining intensity* by the *grade*. The staining intensity is scored on a scale 0-3, where 0 means *no staining*, 1+ *weak*, 2+ *moderate* and 3+ *high*, and is determined on the basis of at least 10 representative fields of 0.125mm^2^ (x500). The grade of gene or protein presence also lies between 0-3: 0 (0-4%), 1 (5-29%), 2 (30-59%), 3 (60-100%).

This scoring step is critical for integration of TMA results. The correlation between biological sequences found in tissues and pathologies is one of the innovations proposed by our system. To integrate information about genes and process involved in different pathologies, firstly we use disease information to cluster TMA experiments into groups of tissues affected by the same disease, independently from the institute they belong to. Then we consider genes and proteins with a high IRS (>4) and relying on GO organizing principles (cellular component, biological process, molecular function) we make statistics of components involved in a specific disease.

Using the developed Perl algorithm we can estimate, for each disease, which are the most visited pathways. While uploading the stained tissue images, the user is asked to specify not only the disease studied but also the biological target of the experiments. The Perl algorithm, relying on GO, tracks all the parents of the indicated target. For every pathology a core database table is created, containing all the GO IDs associated to a counter, which is increased every time the ID is involved in the ascending ontology path to the root term. The values of these variables are visualized in the web site as bar lines of proportional length. Statistics are visualized in a shared page of the web site that can be freely accessed by all authenticated users. The ontological approach of these statistics, which relies on GO categories, allows the identification of biologically consistent linkage between specific pathways and the examined pathology. This aspect is crucial for a deeper understanding of the biomolecular mechanisms mainly related to every specific cancer type and provides an effective integration of bioinformatics data in pathological analysis.

It is worthy to note that the use of conventionally defined terms also provides information about research topics, and consequent linking of papers related to the same context. When a page of our site displaying sample information is shown, the ontology terms are hyperlinked through the MeSH system and the user can easily access published papers concerning the same topic.

### Availability

The TMA management system can be accessed through the World Wide Web using any recent web browser. URL: 

## Conclusions

The developed infrastructure is useful in *pre* TMA construction (sample description level), allowing the uploading of HE reacted sample images followed by an ontology description to better identify the kind of tissue, and in the *post* construction (after TMA has been physically built), giving the possibility of adding information about genes or proteins searched on tissues and of performing statistical analysis on these data, allowing data integration between different cancer types and specific gene or protein presence. The innovative feature of ontology based description for tissue origin, morphology and staining, provides a complete and community-recognized identification of samples, which, according to us, is crucial for tissue sharing. The availability of ontological terms allows the development of semantic web searches in papers contained in the PubMed repository, thus improving data integration.

A first attempt to add bioinformatics methods to TMA analysis is represented by the study of the most involved molecular pathways.

Users of this database will be the pathologists of medical institutions which can archive data related of their preserved tissues, and the members of the scientific community who are interested in creating their own TMA use cases. The system represents a place where different competences and abilities can meet, moving towards biological sample sharing, in order to better design TMA experiments and obtain interesting and reliable results. Sharing is, in fact, the only way to build rich and complete TMA blocks collections, to verify researcher's expectations about the biomolecular content of cancer tissues.

The groups which are involved in this project are the Institute for Biomedical Technologies of the National Council for Research, Biorep s.r.l, a company which specializes in biomaterial nitrogen based storage, and Multimedica, a private hospital in where there is an active anatomo-pathology division. A previous additional contribution came from National Cancer Institute (INT, Milan, Italy), which participated to metadata definition and interface organization.

## List of abbreviations used

AJAX: Asynchronous JavaScript and XML

GO: Gene Ontology

GOA: Gene Ontology Annotation

HE: Hematoxylin-Eosine

HTTP: HyperText Transfer Protocol

HTTPS: Secure HTTP

ID: IDentifier

INT: National Institute for Tumor

IRS: ImmunoReactive Score

MeSH: Medical Subject Heading

NCBI: National Center for Biotechnology Information

NIH: National Institutes of Health

OBO: Open Biomedical Ontology

OLS: Ontology Lookup Service

PEAR: PHP Extension and Application Repository

PHP: Hypertext Preprocessor

RDBMS: Relational DataBase Management System

SNOMED: Systematized NOmenclature of MEDicine

SOAP: Simple Object Access Protocol

TCP: Transmission Control Protocol

TMA: Tissue MicroArray

WSDL: Web Service Definition Language

## Competing interests

The authors declare that they have no competing interests.

## Authors' contributions

FV designed the database, the data integration system and the web interface. IM was involved in the technical aspects of the implementation and in the database definition. AC, BL and AS designed and developed the GO link system. LM coordinates the database specification and the development of the full text literature mining interface. All authors read and approved the final manuscript.
